# Supplementary material for: Levels and determinants of quality antenatal care in Bangladesh: Evidence from the Bangladesh Demographic and Health Survey
Source: PLoS One. 2023 May 3;18(5):e0269767. doi: 10.1371/journal.pone.0269767 (PMC10155983; doi:10.1371/journal.pone.0269767)
Supplement: S1 File — (DOCX) [file pone.0269767.s001.docx]

**S1 Table: Factors associated with quality of antenatal care received (full results of the un-adjusted regression analysis)**

| **Background**  **characteristics** | **Middle-quality ANC** | | | **High-quality ANC** | | |
| --- | --- | --- | --- | --- | --- | --- |
|  | **Crude Odds Ratio**  **(95% Confidence Interval)** | **Standard error** | **P-value** | **Crude Odds Ratio**  **(95% Confidence Interval)** | **Standard error** | **P-value** |
| **Survey year** | | | | | | |
| 2014 | 1.00 |  |  | 1.00 |  |  |
| 2017–18 | 1.12 (1.01–1.24) | 0.060 | 0.037 | 2.13 (1.91–2.37) | 0.117 | <0.001 |
| **Division** | | | | | | |
| Dhaka | 1.00 |  |  | 1.00 |  |  |
| Barisal | 1.34 (1.05–1.70) | 0.163 | 0.018 | 0.80 (0.62–1.03) | 0.103 | 0.086 |
| Chittagong | 1.33 (1.14–1.54) | 0.100 | <0.001 | 0.85 (0.73–0.98) | 0.064 | 0.028 |
| Khulna | 1.13 (0.92–1.38) | 0.115 | 0.237 | 1.05 (0.86–1.27) | 0.102 | 0.647 |
| Rajshahi | 0.82 (0.68–0.98) | 0.077 | 0.032 | 0.65 (0.54–0.78) | 0.060 | <0.001 |
| Rangpur | 0.87 (0.72–1.05) | 0.083 | 0.139 | 0.66 (0.55–0.80) | 0.063 | <0.001 |
| Sylhet | 1.27 (1.03–1.57) | 0.137 | 0.025 | 0.74 (0.59–0.92) | 0.084 | 0.007 |
| Mymensingh | 0.66 (0.51–0.87) | 0.092 | 0.003 | 0.67 (0.52–0.86) | 0.085 | 0.002 |
| **Place** **of residence** | | | | | | |
| Urban | 1.00 |  |  | 1.00 |  |  |
| Rural | 0.74 (0.65–0.84) | 0.047 | <0.001 | 0.36 (0.32–0.41) | 0.021 | <0.001 |
| **Wealth quintile** | | | | | | |
| Richest | 1.00 |  |  | 1.00 |  |  |
| Richer | 0.64 (0.53–0.77) | 0.060 | <0.001 | 0.31 (0.26–0.37) | 0.027 | <0.001 |
| Middle | 0.40 (0.33–0.48) | 0.037 | <0.001 | 0.15 (0.13–0.18) | 0.014 | <0.001 |
| Poorer | 0.38 (0.31–0.45) | 0.035 | <0.001 | 0.10 (0.08–0.12) | 0.009 | <0.001 |
| Poorest | 0.30 (0.25–0.36) | 0.028 | <0.001 | 0.06 (0.05–0.07) | 0.006 | <0.001 |
| **Education level** | | | | | | |
| Higher | 1.00 |  |  | 1.00 |  |  |
| Secondary | 0.55 (0.45–0.66) | 0.052 | <0.001 | 0.26 (0.22–0.30) | 0.022 | <0.001 |
| Primary | 0.42 (0.34–0.50) | 0.041 | <0.001 | 0.10 (0.08–0.12) | 0.009 | <0.001 |
| No education | 0.28 (0.22–0.36) | 0.036 | <0.001 | 0.06 (0.04–0.08) | 0.009 | <0.001 |
| **Age in years** | | | | | | |
| 15–19 | 0.95 (0.79–1.14) | 0.087 | 0.577 | 0.81 (0.68–0.98) | 0.076 | 0.027 |
| 20–24 | 1.07 (0.91–1.26) | 0.090 | 0.413 | 1.15 (0.97–1.35) | 0.096 | 0.104 |
| 25–29 | 1.03 (0.86–1.22) | 0.090 | 0.762 | 1.14 (0.96–1.35) | 0.100 | 0.137 |
| 30–34 | 1.00 |  |  | 1.00 |  |  |
| 35–49 | 0.86 (0.67–1.12) | 0.116 | 0.277 | 0.98 (0.76–1.27) | 0.129 | 0.897 |
| **Birth order** | | | | | | |
| 1 | 1.00 |  |  | 1.00 |  |  |
| 2–3 | 0.88 (0.78–0.98) | 0.050 | 0.021 | 0.73 (0.65–0.81) | 0.041 | <0.001 |
| 4–5 | 0.58 (0.48–0.70) | 0.055 | <0.001 | 0.31 (0.25–0.38) | 0.033 | <0.001 |
| 6+ | 0.60 (0.41–0.88) | 0.117 | 0.009 | 0.22 (0.13–0.38) | 0.059 | <0.001 |
| **Religion** | | | | | | |
| Islam | 1.00 |  |  | 1.00 |  |  |
| Others | 1.40 (1.15–1.71) | 0.142 | 0.001 | 1.54 (1.27–1.87) | 0.152 | <0.001 |
| **Exposed to media** | | | | | | |
| Exposed | 1.00 |  |  | 1.00 |  |  |
| Unexposed | 0.61 (0.55–0.68) | 0.033 | <0.001 | 0.27 (0.25–0.31) | 0.016 | <0.001 |

**S2 Table: Factors associated with quality of antenatal care received (full results of the adjusted regression analysis)**

| **Background**  **characteristics** | **Middle-quality ANC** | | | **High-quality ANC** | | |
| --- | --- | --- | --- | --- | --- | --- |
|  | **Adjusted Odds Ratio**  **(95% Confidence Interval)** | **Standard error** | **P-value** | **Adjusted Odds Ratio**  **(95% Confidence Interval)** | **Standard error** | **P-value** |
| **Survey year** | | | | | | |
| 2014 | 1.00 |  |  | 1.00 |  |  |
| 2017–18 | 1.23 (1.10–1.37) | 0.070 | <0.001 | 2.70 (2.38–3.06) | 0.174 | <0.001 |
| **Division** | | | | | | |
| Dhaka | 1.00 |  |  | 1.00 |  |  |
| Barisal | 1.63 (1.27–2.08) | 0.207 | <0.001 | 1.37 (1.03–1.83) | 0.201 | 0.032 |
| Chittagong | 1.31 (1.12–1.53) | 0.103 | 0.001 | 0.87 (0.73–1.03) | 0.076 | 0.100 |
| Khulna | 1.17 (0.95–1.45) | 0.125 | 0.133 | 1.25 (1.01–1.56) | 0.141 | 0.045 |
| Rajshahi | 0.87 (0.72–1.05) | 0.086 | 0.151 | 0.79 (0.64–0.98) | 0.085 | 0.028 |
| Rangpur | 1.04 (0.85–1.27) | 0.106 | 0.699 | 1.14 (0.91–1.42) | 0.128 | 0.253 |
| Sylhet | 1.53 (1.23–1.91) | 0.173 | <0.001 | 1.25 (0.96–1.62) | 0.165 | 0.093 |
| Mymensingh | 0.73 (0.55–0.97) | 0.106 | 0.031 | 0.80 (0.60–1.07) | 0.119 | 0.138 |
| **Place** **of residence** | | | | | | |
| Urban | 1.00 |  |  | 1.00 |  |  |
| Rural | 1.05 (0.91–1.21) | 0.076 | 0.511 | 0.78 (0.67–0.90) | 0.058 | 0.001 |
| **Wealth quintile** | | | | | | |
| Richest | 1.00 |  |  | 1.00 |  |  |
| Richer | 0.70 (0.57–0.84) | 0.068 | <0.001 | 0.41 (0.34–0.49) | 0.039 | <0.001 |
| Middle | 0.44 (0.36–0.54) | 0.046 | <0.001 | 0.22 (0.18–0.27) | 0.023 | <0.001 |
| Poorer | 0.46 (0.37–0.57) | 0.051 | <0.001 | 0.17 (0.13–0.21) | 0.020 | <0.001 |
| Poorest | 0.40 (0.31–0.50) | 0.048 | <0.001 | 0.13 (0.10–0.17) | 0.017 | <0.001 |
| **Education level** | | | | | | |
| Higher | 1.00 |  |  | 1.00 |  |  |
| Secondary | 0.72 (0.60–0.88) | 0.073 | 0.001 | 0.52 (0.43–0.62) | 0.049 | <0.001 |
| Primary | 0.66 (0.53–0.81) | 0.072 | <0.001 | 0.27 (0.21–0.33) | 0.030 | <0.001 |
| No education | 0.48 (0.36–0.64) | 0.069 | <0.001 | 0.20 (0.15–0.28) | 0.034 | <0.001 |
| **Age in years** | | | | | | |
| 15–19 | 0.71 (0.56–90) | 0.086 | 0.004 | 0.60 (0.46–0.77) | 0.079 | <0.001 |
| 20–24 | 0.77 (0.56–0.90) | 0.076 | 0.007 | 0.70 (0.56–0.87) | 0.077 | 0.001 |
| 25–29 | 0.87 (0.72–1.05) | 0.083 | 0.137 | 0.92 (0.75–1.13) | 0.097 | 0.446 |
| 30–34 | 1.00 |  |  | 1.00 |  |  |
| 35–49 | 1.00 (0.76–1.33) | 0.143 | 0.980 | 1.41 (1.03–1.92) | 0.222 | 0.031 |
| **Birth order** | | | | | | |
| 1 | 1.00 |  |  | 1.00 |  |  |
| 2–3 | 0.84 (0.72–0.97) | 0.064 | 0.021 | 0.69 (0.59–0.81) | 0.056 | <0.001 |
| 4–5 | 0.55 (0.43–0.71) | 0.070 | <0.001 | 0.36 (0.27–0.48) | 0.054 | <0.001 |
| 6+ | 0.67 (0.43–1.06) | 0.154 | 0.085 | 0.45 (0.25–0.82) | 0.138 | 0.009 |
| **Religion** | | | | | | |
| Islam | 1.00 |  |  | 1.00 |  |  |
| Others | 1.34 (1.09–1.65) | 0.142 | 0.005 | 1.39 (1.11–1.73) | 0.158 | 0.004 |
| **Exposed to media** | | | | | | |
| Exposed | 1.00 |  |  | 1.00 |  |  |
| Unexposed | 0.84 (0.74–0.95) | 0.053 | 0.005 | 0.65 (0.56–0.75) | 0.046 | <0.001 |
