# Supplementary material for: Levels and determinants of quality antenatal care in Bangladesh: Evidence from the Bangladesh Demographic and Health Survey
Source: PLoS One. 2023 May 3;18(5):e0269767. doi: 10.1371/journal.pone.0269767 (PMC10155983; doi:10.1371/journal.pone.0269767)
Supplement: Attachment — Submitted filename: Response to Reviewers.docx [file pone.0269767.s002.docx]

**To**

**The Editor,**

**PLOS ONE**

Dear Editor,

Thank you very much for your time and allowing us to submit our revised manuscript titled **“Levels and determinants of quality of antenatal care in Bangladesh: Evidence from the Bangladesh Demographic and Health Survey”** in your reputed journal. We have tried our best to address all of the reviewer’s comments accordingly.

Sincerely yours,

Ema Akter

Maternal and Child Health Division, icddr,b (International Centre for Diarrhoeal Disease Research, Bangladesh), Dhaka, Bangladesh

//////////////////////////////////////////////////////////////////////////////////////////////////////////////

**Reviewers' comments:**

**Comments to the Author**

Abstract: fine no comments from me. Please rewrite the conclusion here it has now a general discussion please be specific based on your findings.

Response: Thank you for your suggestion. We have updated it as “Although there has been an improvement in the quality of ANC from 2014 to 2017/18, it is still of poor standard in Bangladesh. There is a need of developing targeted interventions for different socio-demographic groups to improve the overall quality of ANC. Future interventions should address both the demand and supply side perspectives.”

Background: please standardised the rationality and novelty of the paper and set the rationality corresponding to other similar studies.

Response: Thanks. We have reviewd the studies related to antenatal care (ANC). There are several studies regarding ANC, of which most of them have been conducted in rural context. Bangladesh Demographic and Health Survey (BDHS) has been conducted every three years which is a nationally representative survey. Also, to our knowledge there is a dearth of study which explored status of quality ANC recently mentioned in BDHS 2017/18. After your suggestion, we have added several statements mentioning how this study contributing to the new knowledge.

“There are several studies on ANC in Bangladesh, of which most of them have been conducted in rural context (24, 29-33). The Demographic and Health Survey has been conducted once every three years, which is a nationally representative survey. Only a few researchers used existing demographic and health survey data to examine the determinants and contents of ANC contacts in Bangladesh (7, 23, 33-36). A study by Rahman et al. (2017) using BDHS 2011 and 2014 explored the determinants of four or more ANC utilisation where the level of education, place of residence and wealth status were found as the determinants (23). Studies using BDHS (2014) to examine the factors associated with ANC, revealed that educated women and women from the richest wealth status were more likely in having ANC (7, 34-36). Another study conducted using BDHS 2007 explored the scenario of ANC services in a rural context and reported that education of the mothers, and being exposed to mass media have a significant effect on having ANC (33). To the best of our knowledge, there is a dearth of study assessing the determinants in receiving the quality of ANC.”

Methods: well organized.

Response: Thank you.

Results l: clear, understandable.

Response: Thank you for your comment.

Discussion: overtly written please be specific when set the logical presentation of the findings relying on relevant literature.

Response: Thanks. We have updated according to your comments in the discussion part.

Conclusion: please sperate recommendations from conclusion. Although it is done based on cross sectional data so recommendations are not necessary.

Response: Thank you. We have updated it.
